# Functional stability analyses of maxillofacial skeleton bearing cleft deformities

**DOI:** 10.1038/s41598-019-40478-w

**Published:** 2019-03-12

**Authors:** Xiangyou Luo, Hanyao Huang, Xing Yin, Bing Shi, Jingtao Li

**Affiliations:** 1 State Key Laboratory of Oral Diseases & National Clinical Research Centre for Oral Diseases, West China Hospital of Stomatology, Sichuan University, 14 Ren Min Nan Road, Chengdu, 610041 China; 2 Department of Oral and Maxillofacial Surgery, West China Hospital of Stomatology, Chengdu, 610041 China; 3 Department of Orthodontics, West China Hospital of Stomatology, Sichuan University, Chengdu, 610041 China

## Abstract

The symmetrically stable craniofacial bony structure supports the complex functions and delicate contour of the face. Congenital craniofacial deformities are often accompanied by bony defects and have been repetitively correlated with compromised dento-maxillary stability, but neither the extent nor the pattern of cleft-related maxillary instability has been explored in detail. Furthermore, it is largely unknown if the bony defect and related instability are correlated with secondary maxillary deformity common among patients with orofacial clefts. With the aid of finite element modeling, we studied the detailed relationship between cleft-related bony defect and maxillary stability under occlusal loading. Craniofacial models were generated based on cone-beam computed tomography data and loaded with mimicked bite forces along the axial axis of each tooth. Our data showed that all cleft models exhibited more asymmetrical deformations under mastication compared with the normal. Models with palatal cleft demonstrated greater asymmetry, greater dental arch contraction, and less maxillary protrusion compared to models with alveolar cleft only. For unilateral cleft models, alveolus on non-cleft side tended to be more protruded and lifted than the cleft side. For bilateral cleft models, the most prominent feature was the seriously contracted alveolar arch and curved and pitched premaxillae. These findings indicated cleft type-specific pattern of maxillary instability, which were largely in accordance with dentoalveolar morphological features among patients. Collectively, our study elucidated the detailed relationship between cleft bony defect and the pattern of maxillary instability, and suggested a prototype for studying the abnormal maxillary and dental arch growth among patients with craniofacial deformities.

## Introduction

Craniofacial bones constitute the most intricate part of human skeletal system. They determine the contour of the face by lending supports and attachments to the overlying soft tissues, and maintain diverse physiological functions in the craniofacial region by providing a stable structure foundation^1,2^. In cases of congenital craniofacial deformities, which are the most common birth defects in human, craniofacial bones are often involved with compromised morphology and function^3,4^. Usually, congenital anomalies affect craniofacial bones in two ways. First, abnormal embryogenesis and development generate primary distortion and defects in the bony structure^5^. Second, the structural instability worsens the primary deformities and skews the growth pattern in a secondary manner^5,6^. The primary distortion and defects could be clearly defined at birth, but the secondary growth abnormalities are still difficult to predict. Although congenital craniofacial deformities have been repeatedly related to the skeletal instability^7–9^, neither the detailed instability pattern nor its correlation with specific tissue defects and growth pattern has been efficiently explored in the previous literature.

Cleft lip and palate is the most common congenital deformity in human dento-craniofacial region, with a varying occurrence of 0.2–2.3‰ in newborns among races^10^. Besides soft tissue anomalies, facial cleft is frequently accompanied by bony defects in the alveolar and palatal regions. The cleft-related maxillary discontinuity often manifests in the form of asymmetry and disproportion^3,4,11^. The midfacial asymmetry coefficient among patients with cleft deformity may reach over 58%^12^, with the distortion most significant in the naso-dento-alverolar region^6,13^.

Moreover, these congenital skeletal deformities tend to become worse during the growth and development after birth. Collapse of maxillary elements adjacent to bony defect is frequently observed among patients, demonstrating narrowed and retracted dental arches, displaced premaxillae, and uneven nasal bases. Such secondary collapse and displacement are often attributed to the “instability” of the disrupted maxillary buttresses^14–20^. Correspondingly, maxillae stabilization has always been listed among the major aims of bone graft management for the cleft defect in the literature^3,21^. The extent and pattern of the cleft-related maxillary instability, as well as the effectiveness of bone graft in stabilizing the maxillae, however, have rarely been illuminated^6,12,22,23^.

In cleft lip and alveolae deformity, the bony defect is limited within the primary palate. When cleft palate occurs simultaneously, the alveolar defect further extends to the posterior and results in complete disruption at the inferior surface of the maxillae. Bilateral and unilateral clefts are also different from each other in separating the maxillae into three and two elements respectively. Although it is convenient to infer that the more extensive the defect the more instable the maxillae, no concrete evidence is available to support this statement or any detailed correlation pattern between cleft severity and maxillae instability^22–24^.

Moreover, along with traumatic surgical intervention and scar contracture, bony defects have been considered as one of the causes for secondary maxillary growth abnormality among patients with clefts^5^. While it is easy to surmise that an instable maxilla tends to be susceptible to pathological influences and easily deviates from normal growth pattern, again, studies either confirming such correlation or illuminating detailed influential patterns were lacking in the literature.

One of the major obstacles to studying the craniofacial skeleton stability is the lack of reliable measuring tools. Some previous studies used artificial instruments to exert forces on cleft alveolus and measured the deformations of the lateral alveolar segments and the premaxillae, but the accuracy of measurement was severely undermined by the poor manoeuvrability of the instrument and individual variations^25,26^. In contrast, with the development of computer technology, non-invasive approaches are now available to analyze the craniofacial structure. The finite element modeling (FEM) has been widely acknowledged as an effective tool^27^ in the biomechanical analyses of craniofacial regions^28^. With accurate modeling from high-resolution CT images and elaborative definition of mechanical loading and boundary conditions, finite element analyses (FEA) could simulate complex functional activities, including the jaw mastication^29^. Therefore, FEM presents an opportunity to study the abovementioned cleft-related maxillary instability.

The most frequent and influential oral activity is the mastication^5^. The deformation characteristics of cleft alveolus under masticatory loading would help us better understand the maxillary instability among patients with CLP.

In this study, we generated finite element models of maxillae affected by different types of cleft defects and analyze their stability and deformation under simulated mastication loadings. The FEA results were also compared with clinical data to further illustrate the potential relationship between maxillary instability and secondary skeletal deformity. Our functional stability analyses serve as a proficient prototype for the study of diverse congenital craniofacial deformities in different clinical settings.

## Materials and Methods

### Ethical approval

Informed consents have been acquired from all the patients (aged 7–15 years old) and their parents for CBCT scanning and analysis of the acquired CBCT images. The research protocol was approved by the ethic committee of West China Hospital of Stomatology, Sichuan University (Approval No. WCHSIRB-D-2016-084R1). All experiments were performed in accordance with the 1964 Helsinki declaration and its later amendments or comparable ethical standards.

### Finite element modeling

**Figure 1:**
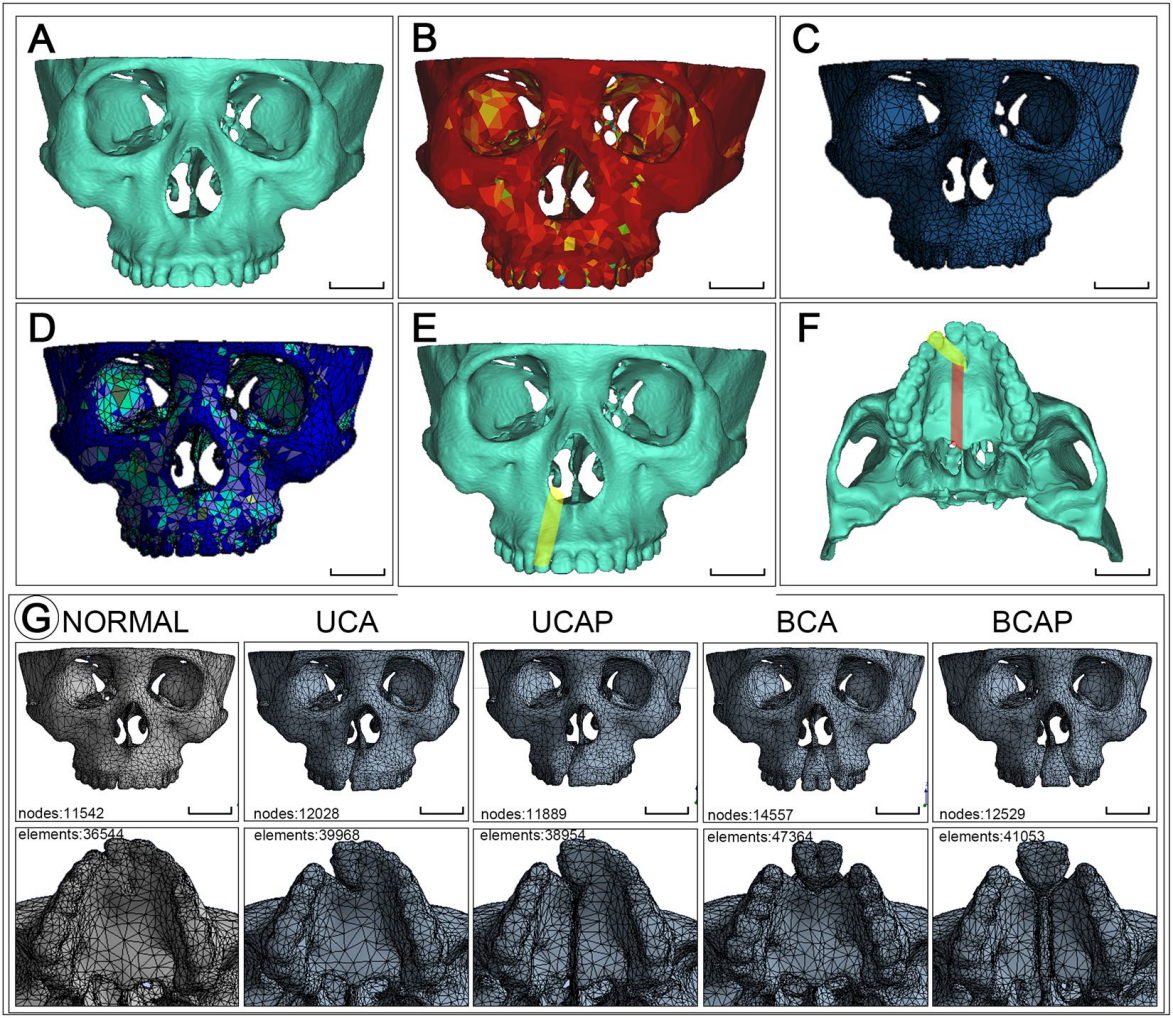
Construction of analytical finite element models. CT images of normal maxilla were three-dimensionally reconstructed in mimics (**A**), after meshed, material properties were assigned to elements based on image Hounsfield values (**B**). Then, meshes (**C**) and materials (**D**) were imported to ANSYS Workbench for biomechanical analysis. Cleft models, for example the unilateral cleft of lip and alveolus and palate (UCAP), were constructed based on the normal model by erasing the corresponding lateral incisor (s) (yellow stripe in (**E**,**F**)), surrounding bone and palatal bone (red stripe in (**F**). Other model construction procedures including meshing and material assigning were kept the same with those of normal model. Except the differences in alveolar/palatal cleft types, cleft models hold great consistency with each other and with the normal model (**G**). Mesh properties including number of nodes and elements are shown in each model pictures (**G**). UCA: unilateral cleft of lip and alveolus; UCAP: unilateral cleft of lip and alveolus and palate; BCA: bilateral cleft of lip and alveolus; BCAP: bilateral cleft of lip and alveolus and palate. Scale bar: 3 cm.

Skull CT images from a Han race Chinese man aged 30 with normal maxillary bony structures, full dentition and well occlusal relationship were obtained from West China Hospital of Stomatology. Images were scanned using Philips MX16^EVO^ machine (PHILIPS N.V., The Netherlands) with tube voltage of 120 kV, tube current of 80 mA and resolution of 0.5 mm. DICOM images were imported into mimics 16.0 and 3-matic 8.0 (Materialise NV, Leuven, Belgium) to be processed and build the normal (control) maxillary FE model. Images were segmented (Hounsfield unit between 226 and 2254), and then 3D reconstructed to build the surface rendering of the whole skull (Fig. 1A). Mandible and certain calvarial and occipital parts of the model were removed. Surface model was then transported into 3-matic and meshed using tetrahedron elements. The solid model with volume meshes was then sent back to mimics. A gray scale based material property algorithm^30^ was applied to generate the elastic modulus and poisson ratio of the maxilla (Fig. 1B).

Then, another four copies of the control model were manually modified to build the cleft models^31^ mimicking UCA (unilateral cleft of lip/alveolus), UCAP (unilateral cleft of lip/alveolus/palate), BCA (bilateral cleft of lip/alveolus), and BCAP (bilateral cleft of lip/alveolus/palate)). For the UCA model, the right lateral incisor was erased together with surrounding buccal and palatal alveolar bone on CT serial images. For UCAP model, further more bone on palatal plate on the right side was removed to simulate conditions in UCAP patients (Fig. 1E,F). For BCA and BCAP models, bone defects on right side in UCA and UCAP models were respectively mirrored to the left side to build bilateral cleft. After clefts were built in each model (Fig. 1G), the succeeding processing procedures were all the same with those of the control model.

### Functional loading and boundary definition

**Figure 2:**
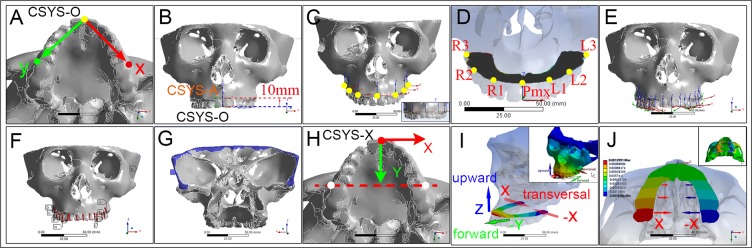
Analytical settings (**A–H**) and results of total deformation in each model (**I–Y**). An occlusal plane coordinate system (CSYS-O) was constructed via three points (**A**) i.e. mesial contact point of central incisors and mesial-buccal cusp peak of bilateral upper first molar. This CSYS-O was offset 10 mm along the Z-axis to achieve the alveolar crest (**B**) and was defined as the coordinate system of alveolar plane (CSYS-A). On the alveolar plane defined by the x- and y- axis of CSYS-A, deformation probes were positioned (**C**) at the central labial/buccal regions of premaxilla (Pmx), canine (L1, R1), first molar (L2, R2) and the third molar (L3, R3) for deformation data collection (**D**). Local coordinate systems were also constructed at the central of occlusal surface of each tooth and z-axis was adjusted to fit the long axis of each tooth (**E**). Simulated masticatory forces were therefore loaded via each local coordinate system through the y-axes guided tooth long axes (**F**). Surfaces along the skull borders were fixed supported for boundary conditions (**G**). In order to depict the 3D deformations of alveolus, another coordinate system (CSYS-X) was defined (**H**) to show the three directions of deformations: x-/y-/z- axis would be separately used to show the transversal/forward-backward/up-down deformations (**I**). X-axis was fit to the line (yellow dotted line and red arrows in H) through the bilateral mesial-buccal cusp peaks of upper first molar. Y-axis was fit to the sagittal plane of the maxilla and cross the occlusal plane. Above settings were kept the same in all models. The alveolar TC gradually increases caudally (**J**).

The volume meshes (Fig. 1C) and material properties (Fig. 1D) of each model were transferred to the modeler component in ANSYS Workbench 15.0 for static structural analysis module. Afterwards, loading and boundary conditions were defined. A local coordinate system at the occlusal surface of each maxillary tooth was established based on the model global coordinate system (Fig. 2E). By offsetting and rotating each local coordinate system, we fit the z-axis along the long axis of each tooth (Fig. 2E). Simulated masticatory forces of 400 N in the molar region, 280 N in the premolar region and 160 N in the anterior region were evenly loaded on the occlusal surface along z-axis of each tooth^32^ (Fig. 2F). The edge of the skull border was fixed supported (Fig. 2G).

### Deformation measurements

To evaluate the deformations of lateral alveolar segments and Pmx, a reference plane (CSYS-A) was defined in the following steps: (1) The occlusal plane^33^ was defined in normal model by appointing its coordinate system (CSYS-O) through the mesial-buccal cusp peak of right/left first molar and the mesial contact point of central incisors (Fig. 2A). (2) This occlusal plane (defined by CSYS-O) was offset for 10 mm to cross the alveolar process and named as CSYS-A (Fig. 2B). (3) The coordinate system data of CSYS-A in normal model was recorded and applied in all cleft models. Seven deformation data probes (Fig. 2C,D) were placed on the reference plane at the buccal alveolae of bilateral canines (R1/L1), first molars (R2/L2), third molars (R3/L3) and the central point of the Pmx. In order to measure the directional deformations of each probe, another coordinate system (CSYS-X) was defined at the mesial contact point of central incisors (Fig. 2H). The X-axis was defined by connecting the mesial-buccal cusp peaks of right/left first molar; and deformations on this direction reflected the transversal contraction of alveolae. The Y-axis was parallel to the occlusal surface and the Z-axis perpendicular to occlusal surface. The Y-axis and Z-axis were respectively defined to detect the protrusion and upward rotation of alveolae or Pmx (Fig. 2I).

To evaluate and compare the degree of deformation asymmetry of maxilla in cleft models with that in control model, the alveolar deformation asymmetry index (AI)^34^ was calculated in each model as follow (min: minimum, max: maximum):

$$\mathrm{AI}\,=[1-\frac{(\frac{{\rm{\min }}(\mathrm{L1},\,\mathrm{R1})}{{\rm{\max }}(\mathrm{L1},\mathrm{R1})}+\frac{{\rm{\min }}(\mathrm{L2},\,\mathrm{R2})}{{\rm{\max }}(\mathrm{L2},\mathrm{R2})}+\frac{{\rm{\min }}(\mathrm{L3},\,\mathrm{R3})}{{\rm{\max }}(\mathrm{L3},\mathrm{R3})})}{3}]\times 100 \% $$

To detect the lateral deformation of alveolae under masticatory loading, transversal contraction (TC) was calculated based on the X-axis deformation of each probe (Rn, Ln, n = 1, 2, 3) (Fig. 2D,I,J) as follow:

${\rm{TCn}}={{\rm{R}}}_{{\rm{n}}}^{{\rm{x}}}-{{\rm{L}}}_{{\rm{n}}}^{{\rm{x}}}$, (n = 1, 2, 3; x refers to deformations on X-axis);

$${\rm{TC}}=({\rm{TC}}1+{\rm{TC}}2+{\rm{TC}}3)/3$$

Deformations on Y-axis direction were recorded as the forward protrusion of alveolus at each probe (green arrow in Fig. 2I). Deformations on Z-axis direction at each probe were calculated as the upward rotation of alveolus (blue arrow in Fig. 2I).

### Cone-beam computed tomography cephalometry

**Figure 3:**
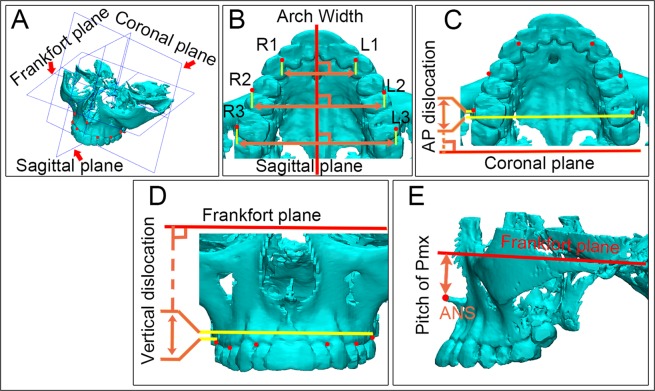
Schemes for CBCT morphometric analysis of alveolus in normal people and alveolar cleft patients. Three reference planes (sagittal plane, FH plane and coronal plane) (red arrows in (**A**) Were respectively defined. Sagittal plane^47^ was defined by crista galli, sellaturcica and basion. Frankfort horizontal plane (FH) was demarcated by 4 points (bilateral Orbitales and bilateral Porions). Coronal plane was set at sellaturcica and perpendicular to the former both planes. Reference points were located bilaterally (red dots in (**B**)) (R: right, L: left) on the mesial-buccal alveolar crest of canine (R1, L1) and second premolar (R2, L2) and the distal-buccal alveolar crest of first molar (R3, L3). Based on each of the three reference planes, transversal, AP (anterior-posterior) and vertical distances of these three group points were measured as the anterior (RI-L1)/middle (R2-L2)/posterior (R3-L3) width (**B**), AP dislocation (**C**) and vertical dislocation (**D**) of the alveolar arch. Distance from the ANS (anterior nasospinale) to the Frankfort plane was measured to scale the pitch of Pmx (**E**), the shorter this distance, the more Pmx was pitched.

Craniofacial Cone-beam computed tomography (CBCT) images of normal person and patients with the four types of cleft deformities aged between 7 and 15 years were enrolled. All enrolled subjects were Chinese Han race. CBCT images of the 50 patients (10 in each group, 5 males and 5 females) were collected. Images were obtained using 3D Accuitomo (J Morita Mfg. Corp., Kyoto, Japan) with radiation dose (85 kV; 4.5 mA) and resolution of 0.25 mm. Dicom formatted Images were segmented with Hounsfield unit between 226–1944 in mimics and surface renderings were then transmitted to 3-matic 8.0 for morphometric analysis. Reference planes including the sagittal plane, Frankfort plane and coronal plane were constructed based on key anatomic points on the skull (red arrows in Fig. 3A). Three groups of probing points (red dots in Fig. 3B,C,D) were located at the mesio-buccal crests of bilateral canines (R1, L1) and second premolars (R2, L2) and the distal-buccal crests of bilateral first molars (R3, L3). Based on the middle sagittal plane, the transversal distances from R1/R2/R3 to L1/L2/L3 were measured as the anterior/middle/posterior width of alveolar arch in each patient (Fig. 3B). Similarly, AP (anterior-posterior)/vertical distances between these three group points were noted as the AP/vertical dislocations of bilateral alveolar segments (Fig. 3C,D). For the calculation of dislocation, we always use distances on non-cleft side to respectively minus those on cleft side in unilateral cleft patients and calculate the absolute values in the normal and patients with bilateral cleft.

The ANS (anterior nasospinale) point was labeled on each maxilla, and its rectangular distance to Frankfort plane was recorded as the pitch of Pmx (Fig. 3E). The shorter this distance was, the more Pmx pitched.

### Statistical analyses

Both FEA and CBCT morphometry were performed by the same investigator (X. L.). Results in each group were recorded as mean ± standard deviation. A Levene’s test was performed to assess the equality of variances for variables calculated in every two groups we compared. A *P-*value above 0.05 was adopted to determine the equal distribution patterns of samples in each two groups. The Student’s independent samples t-test was used to test the significance of difference described in this article by using the SPSS 19.0 software (IBM Corporation, NY, USA). *P* < *0.05* was considered statistically significant.

## Results

### Cleft maxillae are asymmetrically deformed under masticatory loading

**Figure 4:**
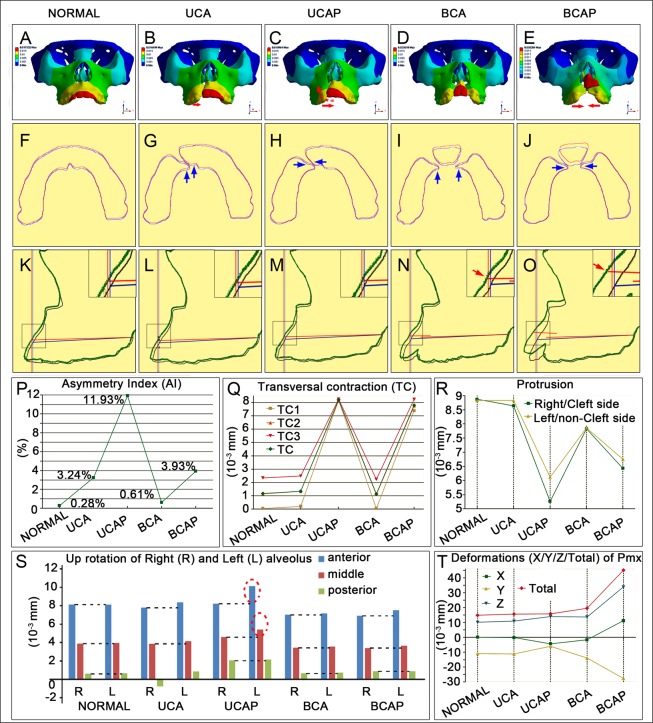
FEA results of total (**A–O**) and directional (**P–T**) deformations show unbalanced and distinctive functional movements in each cleft model. Frontal (**A–E**), sagittal (**F–J**) and transversal (**K–O**) views of deformed alveolus in each model were plotted. Gray contour lines in F-J and blue contour line in K-O show the shape of each maxilla before loaded. Under normal mastication, Alveolus in normal showed mild contraction (**K**,**Q**), protrusion (**K**,**R**) and lifting (**F**,**S**), these deformations were quite symmetrical (**P**). On X direction, palatal cleft models show much more seriously contracted alveolar arch than others (**Q** and red arrows in **B–E**, blue arrows in **M**,**O**). On Y direction, simple alveolar cleft models were relatively more protruded than palatal cleft models (**R** and blue arrows in **L**,**N**), though protrusions in cleft models were all smaller than those in normal. Protrusion on noncleft side was larger than that on cleft side in UCA and UCAP (**R**). On Z direction, alveolar liftings in bilateral cleft models were smaller than those in normal, liftings in noncleft side were larger than the cleft side in unilateral cleft models especially UCAP (red dotted circles in **S**). For Pmx, it was remarkably protruded and lifted (**D**,**E**,**I**,**J**) with greatly increased total deformations in BCA and BCAP (**T**). In UCAP, Pmx showed lateral inclination and rotation toward the cleft side (**C**,**T**).

The deformation simulated in each model was illustrated with a color-coded scale (Fig. 4A–E). Deformation at each specific anatomic element on the monitoring plane (defined by CSYS-A in Fig. 2B) was quantified. The overall symmetry of the mastication-generated deformation was evaluated using asymmetry index (AI). The asymmetry was minimal in the normal control (AI = 0.28%), and slightly increased in the BCA model (AI = 0.61%). When it came to models with palatal cleft, the asymmetry was significantly worsened (Fig. 4P). Generally, the unilaterality of the cleft and the presence of palatal defect seemed to be correlated with the asymmetry in the deformation.

### Maxillae suffering palatal cleft are susceptible to dental arch collapse

The simulated deformations under masticatory loading demonstrated some shared characteristics among the models, including transversal collapse of the dental arch toward the middle line (Fig. 4Q), anterior protrusion (Fig. 4R), and counter-clock rotation (Fig. 4S).

The extent of transversal dental arch collapse, as measured at the levels of canine (TC1), premolar (TC2), and molar (TC3), was minimal and comparable among the normal, UCA and BCA models, but greatly increased in models with palatal cleft (Fig. 4Q, red arrows in Fig. 4C,E and blue arrows in H,J). In addition, in models without cleft palate, the extent of transversal collapse was greater at the posterior portion than the anterior (Fig. 4Q). In models with cleft palate, the collapse extent was about the same at the three measuring levels (Appendix Table 1).

### Mastication-driven protrusion of the maxillae is reduced by the presence of cleft deformities

Masticatory loading led to protrusion in the sagittal plane in all models (Fig. 4R). Compared with the normal, the extent of anterior protrusion was smaller when the cleft deformities were present (Fig. 4R, Appendix Table 2), especially when the cleft involved the palate. The least protrusion was observed in the UCAP model. For models with unilateral cleft, the cleft side segment was less protrusive than the non-cleft side (Fig. 4R). For bilateral cleft models, protrusion on both sides was similar (Fig. 4R). Collectively, models with palatal cleft presented more severe transversal arch collapse and less sagittal protrusion than other models (blue arrows in Fig. 4G–J).

### Bony segments in unilateral cleft maxillae rotate unevenly

On the Z direction, axial loaded bite forces also rendered upward deformations to each alveolar segment (clock-wise rotation on the sagittal plane when viewed from the left) (Fig. 4K–O). Such upward rotation in the normal model was symmetric and its extent gradually increased from posterior to anterior (Fig. 4S). In the cleft models, however, the rotation stimulated by mastication loading was asymmetric between cleft segments, especially with the presence of palatal defect. Interestingly, among unilateral cleft models, the upward rotation was greater on the non-cleft side than on the cleft side (red dotted circles in Fig. 4S). The upward rotation of the lateral maxillary segments in bilateral cleft models was smaller when compared to the normal model (Fig. 4S). Unexpectedly, the posterior alveolus at the cleft side of the UCA model demonstrated downward movement (−0.79 × 10^−3^mm) under masticatory loading (Fig. 4S).

### The pattern of the premaxillae instability is cleft-type dependent

The total deformation magnitudes of the Pmx segment were significantly higher in the BCA and BCAP models when compared with the others (red arrows in Fig. 4N,O, and red line in Fig. 4T, Appendix Table 3). Greater upward rotation and cleft-side deviation of the Pmx were observed in the UCAP than in the UCA model (red dotted arrow in Fig. 4C). When compared with the normal, the models with bilateral clefts demonstrated greater Pmx protrusion, while those with unilateral demonstrated smaller protrusion (yellow line in Fig. 4T). The BCA model demonstrated less horizontal deviation and upward rotation while more protrusion when compared with the UCAP models. The BCAP model demonstrated the most severely dislocated Pmx (Fig. 4E,J,O), which held the highest magnitude of deformations on all directions (Fig. 4T; Appendix Tabel 3).

### FEA simulations coincide with the clinical manifestation of secondary cleft deformities of the maxillae

**Figure 5:**
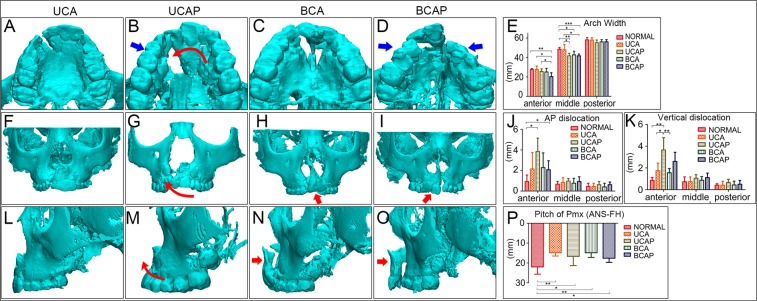
Results of CBCT morphometric analysis of four kinds of alveolar cleft patients. (**A–D**) shows the transversal views of representative patient’s alveolus and the average alveolar width in each cleft type (**E**). Alveolar width was significantly reduced in palatal cleft patients (blue arrows in **B**,**D**). Alveolar AP dislocations were found increased in all cleft type patients, especially in palatal cleft patients (**J**). Vertical dislocations were also mostly observed in palatal cleft patients particularly in UCAP. Pmx was significantly pitched in all cleft patients (red arrows in **H**,**I**,**N**,**O**,**P**). Typical inclination and rotation of alveolus on non-cleft side toward the cleft was observed (curved red arrows in **B**, **G**, and in **M**). ^*^P < 0.05, ^**^P < 0.01, ^***^P < 0.001.

CBCT cephalometry was performed among clinical patients to confirm the FEA results. The anterior arch width was significantly smaller among patients with BCAP (20.13 ± 4.01) when compared with the other three groups (normal [27.57 ± 0.96; *P* = 0.004], UCA [27.7 ± 3.45; *P* = 0.013] and UCAP [25.52 ± 2.77; *P* = 0.038]). The middle arch width in patients with cleft palate was significantly smaller than in the normal and patients without palatal cleft. The middle alveolar width in UCAP (41.56 ± 2.75) was narrower than those of normal (48.5 ± 1.87; *P* = 0.002) and UCA (47.93 ± 5.28; *P* = 0.044); middle alveolar width of BCAP (41.75 ± 1.9) was also smaller than those of normal (48.5 ± 1.87; *P* = 0.000) and UCA (47.93 ± 5.28; *P* = 0.039). The arch width measurements indicated that palatal cleft tended to cause narrower dental arch at the anterior and middle third of the palate (Fig. 5E), which coincides with the FEA results that palatal cleft led to remarkable alveolar transversal contraction (Fig. 4Q).

On the AP dimension, greater discrepancy between the bilateral maxillary elements were observed among patients with palatal cleft (greater AP dislocation in UCAP [3.84 ± 1.31; *P* = 0.016] and BCAP [2.07 ± 0.88; *P* = 0.037] groups than the normal [0.92 ± 0.64]), which was consistent with the protrusion simulation in our FEA models (compare 5J and 4R). On the vertical dimension, the highest discrepancy was observed in the UCAP (3.65 ± 1.13) group which is significantly greater than the normal (0.86 ± 0.27; *P* = 0.001) and the UCA (1.75 ± 0.69; *P* = 0.012) and BCA (1.56 ± 0.39; *P* = 0.004) groups. Interestingly, these significant differences were all concentrated in the anterior alveolar regions, showing that alveolar segments on the non-cleft side were always more anteriorly and upwardly located than the segments on the cleft side. This is consistent with our FEA results that UCAP model showed remarkable bilateral discrepancies on AP (Fig. 4R) and vertical (Fig. 4S) dimensions. The inclination and rotation of the larger noncleft side segment (together with Pmx) towards the cleft side in UCAP group observed in CBCT images (red curved arrows in Fig. 5B,G,M) also matched the FEA results (red dotted arrow in Fig. 4C).

Furthermore, the pitch (or the upward rotation as demonstrated by the distance between ANS and FH) of the premaxillae was significantly greater among UCA [14.89 ± 1.63; *P* = 0.004], UCAP [16.7 ± 4.63; *P* = 0.046], BCA [14.89 ± 2.35; *P* = 0.007], and BCAP [17.63 ± 2.11; *P* = 0.049] patients than among the normal [22 ± 3.67] (Fig. 5P and red arrows in Fig. 5H,I,N,O), which was also indicated in FEA simulated movements on Z-axis (Fig. 4T). In general, the deformations suggested by the CBCT cephalometry among patients with clefts are largely consistent with those predicted by our FEA simulations.

## Discussion

Intact midfacial bones form the three-dimensional biomechanical buttresses^35^. These cross-linked buttresses constitute a strong mechanical grid to disperse masticatory forces from teeth to the skull^32^, keeping the maxillae stable^36^. Previous studies revealed that the palatoalveolar complex played a significant role in the maxillofacial buttressing^37^. On the vertical direction, all vertical buttresses begin at the alveolar process. Therefore, the integrity of alveolar process makes vertical loads distribute evenly among the media, lateral and posterior buttresses. On the horizontal direction, the palatoalveolar complex forms the major part of the middle horizontal buttress^36^ and its support maintains the U-shaped configuration of upper dentition. On the sagittal (AP) direction^37^, the hard palate articulated through the palatine bones with the pterygoid plates of the sphenoid bone, preventing AP dislocation of lateral alveolae. Thus, interruption in palatoalveolar complex from fractures^2,35,37^ or congenital diseases may lead to collapse of the mid-facial buttresses and result in deformations.

Among patients with congenital cleft deformities, the supporting matrix of the maxillae is disrupted by the bony defects at the alveolae^21^ and the palate^26^. Although cleft-related maxillary instability has been repeatedly mentioned in previous literature, its detailed pattern and consequences in varying types of cleft deformities has rarely been studied^3,25,26^.

One of the major obstacles to in-depth analyses of cleft-related maxillary instability used to be methodological. No capable tool is available to physically measure the stability of the maxillae. Stenstroem SJ, etc.^38,39^ proposed a device anchored onto the dentition or alveolae with studs, and measured the stability of the maxillae by reading the range of movement under certain pushing force. Beside its untested accuracy, this device could not assess multidirectional deformation of the maxillae under more complicated intraoral force loadings.

Fortunately, the development of digital modeling technology lends us a tool to recapitulate the cleft maxillae and masticatory loading with more accuracy. For its low risk, high efficiency and validity, FEM has been successfully applied in approaching craniofacial problems including bone fracture reduction and rhinoplasty^40^.

In the present study, four types of cleft FE models were generated from the CT data of a normal subject. Except the type of alveolar/palatal clefts, all procedures including CT image reconstruction, surface rendering, meshing and material assigning were kept the same to eliminate individual deviation^41^. Since the material properties were assigned according to the gray values in CT images, the different modulus of bones in various mineralization extent was simulated. As all models were constructed from the same CT images, local coordinate systems for simulated bite force loading, occlusal plane placing and deformation probes locating were built following completely same protocol. All the above settings served the purpose of maintaining both the accuracy and comparability among the models.

The most significant physiological mechanical loading to the maxillae is from mastication. Masticatory force, though of smaller magnitude than traumatic forces, is constantly transmitted via teeth and shapes the growth and remodeling of the maxillae^2^. Alterations on masticatory force or occlusal relationship may cause uneven distribution of biological strains or deformations, and lead to skeletal growth deformities^42^. For instance, unilateral mastication has been found to cause facial asymmetry^43^. Similarly, bony discontinuity and tooth loss of cleft maxillae would alter the distributive pattern of masticatory force and consequently the final biomechanical and growth presentation of the maxillae. Again, such complicated biomechanical situations could not be physically evaluated with ease.

A structurally intact maxilla renders balanced and stable biomechanical supports, bearing masticatory forces^2^ transmitted via tooth roots to alveolus. In normal maxilla, bony structures and masticatory forces are quite symmetrical for functional stability. Intact maxilla also shows strong resistance to unbalanced deformation. Alveolar deformations are highly symmetrical (with AI of 0.28% shown in Fig. 4P) on the left and right side.

However, as observed in our results, AI varies greatly among different cleft types (Fig. 4P). In bilateral cleft models, though bony structures were still symmetrical, alveolar/palatal clefts compromised the biomechanical resistance of maxilla. The more extent the bone loss, the more instable the maxilla becomes (AI: BCAP > BCA) (Fig. 4P). For the unilateral cleft models, both bony frames and bite forces were asymmetrical. These contribute to the seriously unstable deformations (AI: UCAP > BCAP, UCA > BCA) (Fig. 4P). These findings indicated that the patterns of maxillary instability, and possibly also the patterns of the disturbed growth, varied with the cleft types.

Alveolar cleft and accompanied tooth loss alter the distributive pattern of bite force and the final biomechanical presentation. For the UCA deformity, reduced anterior bite force on cleft side and broken alveolar integrity lead to less protrusion on cleft side (Fig. 4R) and more anterior lifting on non-cleft side (Fig. 4S). For the UCAP, thoroughly break-up of alveolus and palate critically destroy the biomechanical support of maxilla. Large alveolar contraction (Fig. 4Q) and retrusion (Fig. 4R) were observed. Effect of unbalanced distribution of bite force was greatly amplified, more retrusion on cleft side (Fig. 4R) and larger lifting on non-cleft side (Fig. 4S) were found. AP and vertical dislocations became prominent (Fig. 5J,K). With the loss of support on the cleft side, Pmx, together with the lateral segment, rotated to the cleft side (Figs 4C,T, 5B,G,M). In the BCA, more reduction on anterior bite force resulted in less alveolar protrusion (Fig. 4R) and lifting (Fig. 4S) than UCA. Loss of bilateral bone support endows the Pmx with large flexibility on all directions (Fig. 4E,T). For BCAP, bilateral thorough clefts made the maxilla feeble and easily deformed (Fig. 4E,T).

As exhibited in the morphological measurement results from the normal and patients with four types of alveolar cleft, many deformity features were found in accordance with the FEA outcome. Significant arch tilting under mastication may finally lead to seriously narrowed alveolar arches^19,44^, particularly among patients with palatal cleft (Fig. 5E). Unbalanced deformations derived from asymmetric biomechanical loading may alter the maxillary growth and contribute to the alveolar dislocations on all directions^45^ (Fig. 5J,K).

Similar to other FEM studies, our analyses bear multiple limitations. Although the bony structure, property assignment, and masticatory loading have been simulated with supreme details, factors including soft tissue coverage, intrinsic growth, and bone remodeling were not fully recapitulated. Mechanical loading was set based on experience data, ignoring more complicated masticatory movements. In addition, the morphology of the models was generated from a normal adult, so that the maxillae ratios and occlusion may be different from those with cleft deformities^46^.

## Conclusion

The stability and deformation of the maxillae bearing various types of cleft deformity was studied under masticatory loading using FEM analyses. Skeletal defects related to congenital maxillofacial deformities lead to significant instability and asymmetry in the deformation during mastication, which coincide to the deviated growth pattern observed clinically. Our study, for the first time, suggests direct relationship among structural defects, functional stability and growth pattern in the craniofacial skeleton, and proposes a reliable prototype to approach the etiology of growth abnormality of diverse congenital craniofacial deformities.

## Supplementary information

Appendix Table 1; Appendix Table 2; Appendix Table 3

Levene's test

T test
