# Supplementary material for: Functional stability analyses of maxillofacial skeleton bearing cleft deformities
Source: Sci Rep. 2019 Mar 12;9:4261. doi: 10.1038/s41598-019-40478-w (PMC6414651; doi:10.1038/s41598-019-40478-w)
Supplement: Supplementary file 1 — Appendix Table 1; Appendix Table 2; Appendix Table 3 [file 41598_2019_40478_MOESM1_ESM.doc]

Functional stability analyses of maxillofacial skeleton bearing congenital deformities

Xiangyou Luo^1,2^, Hanyao Huang^1,2^, Xing Yin^1,3^, Bing Shi^1,2^, Jingtao Li^1,2,*^

1. State Key Laboratory of Oral Diseases & National Clinical Research Centre for Oral Diseases, West China Hospital of Stomatology, Sichuan University, 14 Ren Min Nan Road, Chengdu, China, 610041.
2. Department of Oral and Maxillofacial Surgery, West China Hospital of Stomatology, Chengdu, China, 610041.
3. Department of Orthodontics, West China Hospital of Stomatology, Sichuan University, Chengdu, China, 610041.

* Correspondence should be addressed to J.Li, lijingtao86@163.com

Appendix Table 1. Transversal dental arch contractions in the anterior (TC1), middle (TC2) and posterior (TC3) part of alveolus and the average magnitude (TC). (Unit: 10^-3^mm).

|  | NORMAL | UCLA | UCLA-P | BCLA | BCLA-P |
| --- | --- | --- | --- | --- | --- |
| TC1 | 0.039 | 0.208 | 8.169 | 0.037 | 7.389 |
| TC2 | 1.144 | 1.334 | 8.166 | 1.103 | 7.749 |
| TC3 | 2.344 | 2.484 | 8.263 | 2.244 | 8.244 |
| TC | 1.176 | 1.342 | 8.199 | 1.128 | 7.794 |

Appendix Table 2. Protrusion of the left and right lateral alveolar segments in each model (Unit: 10^-3^mm) and the percentage of alveolar protrusion in cleft models compared to that in normal model.

|  | NORMAL  Value (%) | UCLA  Value (%) | UCLA-P Value (%) | BCLA Value (%) | BCLA-P  Value (%) |
| --- | --- | --- | --- | --- | --- |
| Right | 8.87(100%) | 8.64(97.4%) | 5.26(59.3%) | 7.83(88.3%) | 6.43(72.5%) |
| Left | 8.83(100%) | 8.83(100%) | 6.12(69.3%) | 7.86(89%) | 6.75(76.4%) |

Appendix Table 3. Three-dimensional deformations of premaxilla. (Unit: 10^-3^mm).

|  | NORMAL | UCLA1 | UCLA1-P | BCLA1 | BCLA1-P |
| --- | --- | --- | --- | --- | --- |
| X | 0.07 | -0.168 | -4.308 | -1.664 | 11.22 |
| Y | -10.904 | -11.108 | -6.048 | -13.88 | -27.657 |
| Z | 10.13 | 10.897 | 13.991 | 13.732 | 33.892 |
| Total | 14.884 | 15.562 | 15.839 | 19.595 | 45.16 |
