# Supplementary material for: Functional stability analyses of maxillofacial skeleton bearing cleft deformities
Source: Sci Rep. 2019 Mar 12;9:4261. doi: 10.1038/s41598-019-40478-w (PMC6414651; doi:10.1038/s41598-019-40478-w)
Supplement: Supplementary file 3 — T test [file 41598_2019_40478_MOESM3_ESM.doc]

Functional stability analyses of maxillofacial skeleton bearing congenital deformities

Xiangyou Luo^1,2^, Hanyao Huang^1,2^, Xing Yin^1,3^, Bing Shi^1,2^, Jingtao Li^1,2,*^

1. State Key Laboratory of Oral Diseases & National Clinical Research Centre for Oral Diseases, West China Hospital of Stomatology, Sichuan University, 14 Ren Min Nan Road, Chengdu, China, 610041.
2. Department of Oral and Maxillofacial Surgery, West China Hospital of Stomatology, Chengdu, China, 610041.
3. Department of Orthodontics, West China Hospital of Stomatology, Sichuan University, Chengdu, China, 610041.

* Correspondence should be addressed to J.Li, lijingtao86@163.com

| dimensional measurements (Mean ± SD)and *P* values between each group (N=Normal) | | | | | | |  |  |  |  |  |  |  |  |  |  |  |
| --- | --- | --- | --- | --- | --- | --- | --- | --- | --- | --- | --- | --- | --- | --- | --- | --- | --- |
|  |  |  |  |  |  |  |  |  |  |  |  |  |  |  |  |  |  |
|  |  | Normal | UCA | UCAP | BCA | BCAP |  | N-UCA | N-UCAP | N-BCA | N-BCAP | UCA-UCAP | UCA-BCA | UCA-BCAP | UCAP-BCA | UCAP-BCAP | BCA-BCAP |
| alveolar width | anterior | 27.57±0.96 | 27.7±3.45 | 25.52±2.77 | 25.05±3.74 | 20.13±4.01 | *P* | 0.938 | 0.157 | 0.182 | 0.004 | 0.304 | 0.278 | 0.013 | 0.825 | 0.038 | 0.080 |
|  | MIDDLE | 48.5±1.87 | 47.93±5.28 | 41.56±2.75 | 42.68±3.75 | 41.75±1.9 | *P* | 0.286 | 0.002 | 0.014 | 0.000 | 0.044 | 0.107 | 0.039 | 0.608 | 0.905 | 0.635 |
|  | POSTERIOR | 58.11±2.32 | 57.91±2.4 | 55.09±2.85 | 56.02±2.33 | 55.76±2.17 | *P* | 0.137 | 0.103 | 0.192 | 0.136 | 0.129 | 0.243 | 0.176 | 0.588 | 0.688 | 0.859 |
| vertical dislocation | ANTERIOR | 0.86±0.27 | 1.75±0.69 | 3.65±1.13 | 1.56±0.39 | 2.6±1.84 | *P* | 0.059 | 0.001 | 0.060 | 0.075 | 0.012 | 0.598 | 0.360 | 0.004 | 0.293 | 0.251 |
|  | MIDDLE | 0.73±0.45 | 0.74±0.43 | 1.06±0.32 | 0.87±0.34 | 1.11±0.42 | *P* | 0.967 | 0.227 | 0.611 | 0.208 | 0.229 | 0.632 | 0.21 | 0.387 | 0.824 | 0.34 |
|  | POSTERIOR | 0.42±0.13 | 0.42±0.36 | 0.66±0.3 | 0.44±0.35 | 0.5±0.35 | *P* | 0.992 | 0.137 | 0.952 | 0.655 | 0.289 | 0.96 | 0.746 | 0.571 | 0.453 | 0.887 |
| A-P dislocation | ANTERIOR | 0.92±0.64 | 2.15±1.59 | 3.84±1.31 | 2.28±1.43 | 2.07±0.88 | *P* | 0.147 | 0.016 | 0.087 | 0.037 | 0.181 | 0.89 | 0.923 | 0.2 | 0.085 | 0.771 |
|  | MIDDLE | 0.67±0.27 | 0.84±0.46 | 0.97±0.25 | 0.77±0.47 | 0.92±0.47 | *P* | 0.641 | 0.346 | 0.79 | 0.497 | 0.598 | 0.817 | 0.793 | 0.426 | 0.846 | 0.628 |
|  | POSTERIOR | 0.45±0.31 | 0.44±0.32 | 0.61±0.32 | 0.41±0.36 | 0.61±0.27 | *P* | 0.977 | 0.446 | 0.863 | 0.386 | 0.435 | 0.886 | 0.377 | 0.388 | 0.967 | 0.338 |
| Pitch of Pmx |  | 22±3.67 | 14.89±1.63 | 16.7±4.63 | 14.89±2.35 | 17.63±2.11 | *P* | 0.004 | 0.046 | 0.007 | 0.049 | 0.434 | 0.999 | 0.051 | 0.459 | 0.693 | 0.089 |
